# Comparative Effectiveness and Safety of Trastuzumab Biosimilars to Herceptin for Adjuvant Treatment of HER2+ Breast Cancer

**DOI:** 10.3390/curroncol31030124

**Published:** 2024-03-21

**Authors:** Caroline Muñoz, Xiaochen Tai, Jessica Arias, Andrea Eisen, Munaza Chaudhry, Scott Gavura, Kelvin K. W. Chan

**Affiliations:** 1 Ontario Health (Cancer Care Ontario), 525 University Ave, Toronto, ON M5G 2L3, Canada; 2 Canadian Centre for Applied Research in Cancer Control, Toronto, ON M5G 2L3, Canada; 3 Sunnybrook Health Sciences Centre, Toronto, ON M4N 3M3, Canada; 4 Temerty Faculty of Medicine, University of Toronto, Toronto, ON M5S 1A8, Canada

**Keywords:** real-world evidence, real-world data, cohort, biosimilar, originator, oncology, breast, treatment, policy, implementation

## Abstract

**Background:** Ontario publicly funds reference trastuzumab (Herceptin) and four biosimilar trastuzumab products for adjuvant treatment of HER2+ breast cancer. We assessed the real-world safety and effectiveness of biosimilar trastuzumab compared to Herceptin for adjuvant treatment of patients with HER2+ breast cancer. **Methods:** This was a population-based, retrospective study comparing the safety and effectiveness of biosimilar trastuzumab and Herceptin for neoadjuvant/adjuvant treatment of HER2+ breast cancer from 2016 to 2021. Treatment patients started biosimilar trastuzumab from November 2019 to June 2021; historical comparator patients started Herceptin from June 2016 to October 2019. Safety outcomes death within 30 days of last dose of trastuzumab, direct hospitalization, emergency department visit leading to hospitalization, early treatment discontinuation, and in-patient admission for congestive heart failure were measured using logistic/negative binomial regression. Overall survival (OS) was measured using Kaplan–Meier methods and Cox proportional hazards regression. Propensity score matching was applied. **Results:** From June 2016 to 2021, 5071 patients with breast cancer were treated with neoadjuvant/adjuvant trastuzumab. The rate of direct hospitalization (RR: 0.85, 95% CI: 0.74–0.98, *p*-value: 0.032) was significantly lower in biosimilar compared to Herceptin patients. OS (log-rank test *p* = 0.98) and risk of mortality (HR: 1.29, 95% CI: 0.72–2.30, *p*-value = 0.39) did not significantly differ between treatment groups. **Conclusions:** Biosimilar trastuzumab demonstrated similar safety and effectiveness to Herceptin. The findings can help improve confidence in and use of biosimilars and demonstrate the value of real-world evidence generation for supporting biosimilar implementations and reassessments.

## 1. Introduction

Breast cancer accounts for approximately 25% of new cancer diagnoses in North America. Amplification of human epidermal growth factor receptor 2 (HER2+)/neu-overexpressing oncogene is present in 20–25% of breast cancers [1]. Trastuzumab (trade name Herceptin, Hoffman-La Roche Ltd., Mississauga, ON, Canada) is a biologic drug used with chemotherapy to treat HER2+/neu-overexpressing adjuvant breast, advanced gastric, gastroesophageal, and esophageal cancers [2]. In Ontario, Canada, trastuzumab plus chemotherapy has been funded for the treatment of adjuvant HER2+ breast cancer by the New Drug Funding Program (NDFP) since 1 August 2005 [3]. Pertuzumab plus trastuzumab for the treatment of adjuvant HER2+ breast cancer was not recommended for reimbursement by the Pan-Canadian Oncology Drug Review (pCODR) Expert Review Committee (pERC) and was subsequently not publicly funded in Ontario [4]. The first biosimilar trastuzumab, Ogivri (BGP Pharma ULC, Dublin 13, Dublin, Ireland), was funded by the NDFP for adjuvant breast cancer in November 2019, followed by Trazimera (Pfizer Canada, Kirland, QC, Canada) later in November 2019, Herzuma (Celltrion Healthcare Co., Ltd., Yeonsu-Gu, Incheon, Republic of Korea) in January 2020, and Kanjinti (Amgen Canada Inc., Mississauga, ON, Canada) in August 2020 [5]. Biosimilar drugs are not exact copies of reference biologic drugs but are highly similar [6]. Biosimilar drugs are not expected to demonstrate clinically meaningful differences in their efficacy and safety compared to reference biologic drugs and undergo the same standard of scientific evaluations by Health Canada prior to authorization [6].

Clinical trials comparing biosimilar and reference trastuzumab have demonstrated similarities in their efficacy and safety [7,8,9,10,11]. A phase 3 randomized controlled trial found similar disease progression and incident treatment-related adverse events and cardiotoxicity among patients with HER2+ early breast cancer receiving trastuzumab biosimilar Herzuma compared to Herceptin [10]. Similarity in effectiveness and cardiac safety has also been demonstrated in a real-world evidence (RWE) study comparing biosimilar Herzuma to Herceptin for the treatment of HER2+ early breast cancer [12]. 

Existing studies have demonstrated comparability between biosimilar and reference trastuzumab; however, these studies may be limited by small sample sizes, study populations with indications different from those in public funding policies, and short follow-up times [7,8,9,10,12]. Real-world studies using administrative data provide an opportunity to explore the effectiveness and safety of biosimilars compared to reference drugs among all patients receiving treatment for their cancer. The findings generated through RWE studies provide stakeholders with additional evidence for assessing new biosimilar policy implementations and shaping future biosimilar policy eligibility criteria and funding details. The purpose of this study was to assess population-based real-world safety and effectiveness of the implementation of biosimilar trastuzumab compared to reference trastuzumab, Herceptin, for adjuvant treatment of patients with HER2+ breast cancer in routine practice.

## 2. Methods

### 2.1. Study Design and Population

This was a population-based, retrospective cohort study comparing the safety and effectiveness of biosimilar trastuzumab to Herceptin among patients with adjuvant HER2+ breast cancer in the real-world setting of Ontario, Canada, using administrative health data housed at Ontario Health (Cancer Care Ontario). All Ontario patients receive universal coverage for publicly funded intravenous oncology drugs administered at out-patient hospitals and cancer centers [3]. 

**Figure 1:**
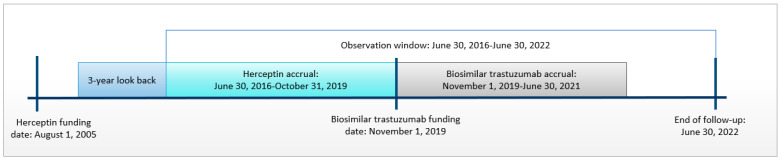
Study design.

Adult patients with HER2+ breast cancer receiving neoadjuvant/adjuvant trastuzumab between 2016 and 2021 were included in the study. Breast cancer diagnosis (ICD-O-3: C50) was identified from the Ontario Cancer Registry (OCR), and receipt of trastuzumab was identified from the NDFP database. The treatment group included patients receiving any biosimilar trastuzumab brand from the start of funding in November 2019 to June 2021. The historical comparator group included patients receiving Herceptin from June 2016 to October 2019. A historical comparator group was used due to the biosimilar trastuzumab funding policy requiring new patients starting treatment with trastuzumab to receive biosimilar as opposed to reference trastuzumab. Herceptin has been used for HER2+ breast cancer since 2005; however, we included only those receiving Herceptin as far back as June 2016 to improve the comparability of treatment patterns received by patients in the treatment and comparator groups (Figure S1). The index date was the date of receipt of the first dose of biosimilar trastuzumab or Herceptin. Patients were followed until 30 June 2022 (Figure 1). 

To be included in this study, all patients must have had a record of trastuzumab within 12 months of breast cancer diagnosis, had a record of neoadjuvant trastuzumab prior to breast resection or adjuvant trastuzumab within 12 months following breast resection, and received systemic chemotherapy within 6 months prior to starting trastuzumab or within 6 months after starting trastuzumab. Only patients who received the following systemic chemotherapy regimens with trastuzumab, as identified by a breast oncologist, were included: anthracycline-based-only regimens (e.g., doxorubicin + cyclophosphamide); anthracycline taxane regimens (e.g., paclitaxel); platinum taxane regimens (e.g., docetaxel plus carboplatin); first-generation cyclophosphamide, methotrexate, fluorouracil (CMF) regimens; second-generation taxane regimens (e.g., docetaxel + cyclophosphamide); and adjuvant weekly paclitaxel and trastuzumab (APT) regimens. Exclusion criteria were as follows: <18 years of age, diagnosis date on or after date of death, missing unique patient identifier or valid population registry record, not a resident of Ontario at the time of diagnosis or treatment, missing age or sex, started trastuzumab prior to provincial funding dates, and received trastuzumab for palliative intent. 

### 2.2. Data Sources

Patients with a breast cancer diagnosis were identified from the OCR, and those with a record of treatment for HER2+ breast cancer treatment were identified from the NDFP database. Dates of death and vital statistics were identified from the Registered Persons Database (RPDB), maintained by the Ministry of Health (MOH). Provincial administrative databases used included the Activity Level Reporting (ALR) systemic therapy and radiation databases, Canadian Institute for Health Information Discharge Abstract Database (CIHI-DAD) and National Ambulatory Care Reporting System (CIHI-NACRS) database, the Ontario Drug Benefit (OBD) out-patient pharmacy claims database, and claims data from the Ontario Health Insurance Plan (OHIP) database. Data elements including baseline characteristics, in-patient and out-patient visits, physician visits, systemic therapy, radiation therapy, and surgery records were extracted from databases and linked between datasets using a unique patient identifier. 

### 2.3. Outcomes

The primary safety outcome was death within 30 days of the last dose of neoadjuvant/adjuvant trastuzumab for HER2+ breast cancer. Secondary safety outcomes included hospital admission from the index date to the date of the last dose of trastuzumab plus 30 days, emergency department (ED) visit resulting in hospital admission from the index date to the date of the last dose of trastuzumab plus 30 days, in-patient hospital admission for congestive heart failure (CHF) defined by a record of in-patient hospital admission data from CIHI-DAD from the index date to the date of the last dose of trastuzumab plus 1 year, and early treatment discontinuation defined by less than 18 trastuzumab treatments. This definition of early treatment discontinuation was chosen to align with NDFP policy that defines the completion of funded trastuzumab treatment as the receipt of 18 treatments for patients receiving a single dose every 3 weeks. The primary effectiveness outcome was overall survival (OS), defined as the time from the index date to death from any cause. Patients were censored on the date of maximum follow-up (30 June 2022).

### 2.4. Covariates

Demographic covariates included age at first trastuzumab treatment and sex from population registry databases. Sociodemographic covariates included Ontario health authorities referred to as Local Health Integrated Networks (LHINs), rural residence, and Ontario Marginalization Index summary score quintiles. These data were obtained using patient postal codes as records at the index date and from the 2016 Canada Census using the Postal Code Conversion File (PCCF+ version 7D). Clinical covariates included stage at diagnosis, history of prior breast cancer, history of prior non-breast cancer, estrogen–progesterone receptor status, Charlson Comorbidity Index scores, CHF diagnosis within 3 years prior to index date, number of days from diagnosis to index date, receipt of neoadjuvant or adjuvant trastuzumab, receipt of neoadjuvant or adjuvant systemic chemotherapy, systemic chemotherapy regimen, receipt of radiation for prior breast cancer, and receipt of radiation for HER2+ breast cancer of interest. Charlson Comorbidity Index scores were calculated for the 3-year lookback window before the index date using CIHI-DAD hospital codes from the most responsible diagnosis, excluding cancer [13].

### 2.5. Statistical Analysis

Baseline characteristics were summarized using descriptive statistics. Continuous variables were reported as means and standard deviations and compared between treatment groups using T-tests for normally distributed variables and non-parametric Wilcoxon rank-sum tests for non-normally distributed variables. Categorical variables were reported as frequencies and percentages and compared between treatment groups using chi-square and Fisher exact tests.

Propensity score matching (PSM) methods were applied to create a PSM cohort with baseline characteristics balanced between treatment groups to mitigate potential confounding as a result of imbalances in the crude cohort [14]. Propensity score matching was applied to estimate the average treatment effect in the population treated with biosimilar trastuzumab. Multivariable logistic regression was used to create propensity scores that estimated a patient’s probability of receiving biosimilar trastuzumab; the multivariable logistic regression included age at first trastuzumab treatment, sex, LHIN, rural residence, Ontario Marginalization Index summary score quintiles, stage at diagnosis, history of prior breast cancer, history of prior non-breast cancer, estrogen–progesterone receptor status, Charlson Comorbidity Index scores, CHF within 3 years prior to index date, number of months from diagnosis to index date, receipt of neoadjuvant or adjuvant trastuzumab, receipt of neoadjuvant or adjuvant systemic chemotherapy, systemic chemotherapy regimen, receipt of radiation for prior breast cancer, and receipt of radiation for HER2+ breast cancer of interest. Patients were hard matched on age at first trastuzumab treatment, sex, stage at diagnosis, estrogen–progesterone receptor status, and systemic chemotherapy regimen. We applied optimal matching with a caliper distance of 0.2, sampled without replacement, and matched 1:2 between biosimilar trastuzumab and Herceptin patients. Standardized differences in baseline characteristics between matched groups of less than 10% indicated acceptable balance. 

The safety outcomes of death within 30 days of the last dose of trastuzumab and in-patient hospital admission for CHF were measured using binomial logistic regression to estimate odds ratios (ORs) and 95% confidence intervals (CIs) for crude and PSM cohorts. Patients with ≥1 year of follow-up data were included in the analysis of in-patient hospital admission for CHF. The safety outcomes hospital admission and ED visit resulting in hospital admission were measured using binomial regression to estimate rate ratios (RRs) and 95% CIs for crude and adjusted cohorts. A separate analysis was conducted for the safety outcome early treatment discontinuation due to our exclusion of patients with a record of trastuzumab emtansine (TDM-1) from this analysis. Patients were able to access TDM-1 for residual early breast cancer after neoadjuvant therapy with trastuzumab and chemotherapy through Compassionate Access Programs and eventually through NDFP policy starting in 2019 [5]. Access to TDM-1 could have resulted in early discontinuation of trastuzumab due to clinicians switching patients from trastuzumab to TDM-1, specifically among the biosimilar group due to the implementation date of TDM-1 being around the biosimilar trastuzumab implementation date. Thus, we chose to exclude patients who switched to TDM-1 to mitigate the potential confounding influence of switching to TDM-1 on the relationship between early treatment discontinuation and treatment exposure. Early treatment discontinuation was measured using binomial logistic regression to estimate ORs and 95% CIs for crude and PSM cohorts. For all safety outcomes, we conducted propensity-score-matched analyses.

In the effectiveness analysis, Kaplan–Meier methods were applied to estimate OS for treatment groups, and the log-rank test was applied to measure the difference in OS between treatment groups for crude and PSM cohorts. Cox proportional hazards regression was used to estimate the risk of mortality, as reported by hazard ratios (HRs) and 95% CIs, for crude and PSM cohorts. A propensity-score-matched analysis was used to estimate the risk of mortality. Statistical significance was <0.05 for all two-sided *p*-values and not adjusted for multiplicity. All analyses were conducted in SAS version 9.4 (SAS Institute, Cary, NC, USA). 

## 3. Results

### 3.1. Study Cohort

**Figure 2:**
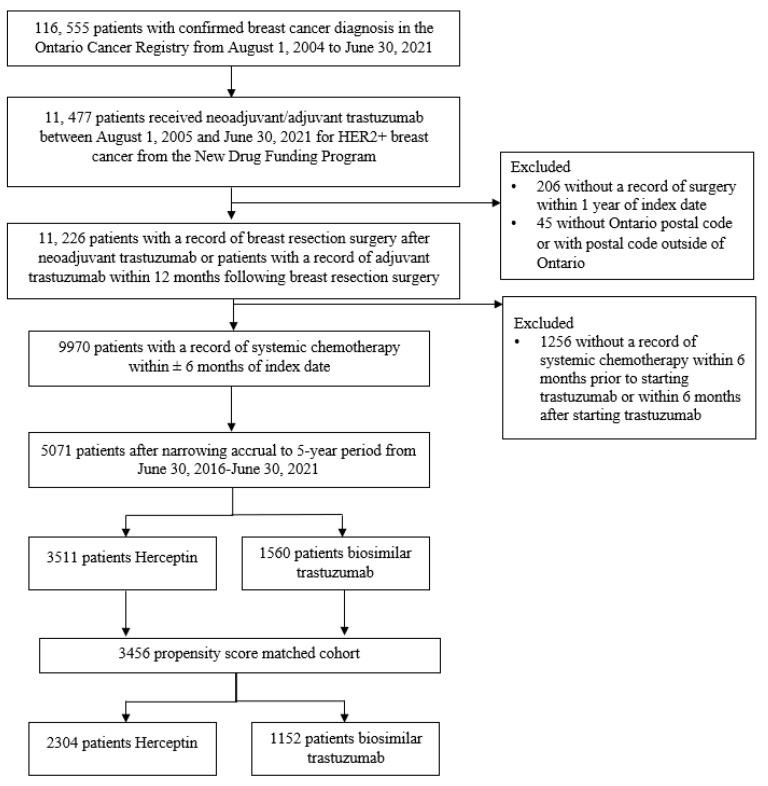
Study cohort.

Between 30 June 2016 and 30 June 2021, a total of 5071 patients with breast cancer received NDFP-funded neoadjuvant or adjuvant trastuzumab biosimilar or Herceptin (Figure 2). Among the 5071 patients, 1560 (31%) received biosimilar trastuzumab and 3511 (69%) received Herceptin. The PSM cohort included 3456 patients matched with a 1:2 ratio of patients receiving biosimilar trastuzumab (n = 1152) to patients receiving Herceptin (n = 2304). Table 1 summarizes the baseline characteristics of the crude and PSM cohorts. All covariates demonstrated standardized differences of <10% indicating a good balance between treatment groups in the PSM cohort. 

### 3.2. Safety Analysis

No significant difference in death within 30 days of the last dose of trastuzumab between treatment groups was identified among the crude cohort (Table 2). The results of the analysis of death within 30 days of the last dose of trastuzumab in the PSM cohort were not reported due to the small number of events identified.

In the PSM cohort, no significant differences were identified between the biosimilar trastuzumab and Herceptin groups for the rate of ED visit leading to hospital admission (RR: 0.96, 95% CI: 0.77–1.20, *p* = 0.72) (Table 2), the odds of in-patient hospital admissions for CHF (OR: 1.12, 95% CI:0.52–2.44, *p* = 0.77) (Table 3), or the odds of early treatment discontinuation (OR: 0.91, 95% CI: 0.75–1.12, *p* = 0.38) (Table 4). A significantly lower rate of direct hospital admission (RR: 0.85, 95% CI: 0.74–0.98, *p* = 0.032) (Table 2) was identified in the biosimilar trastuzumab group. 

### 3.3. Effectiveness Analysis 

**Figure 3:**
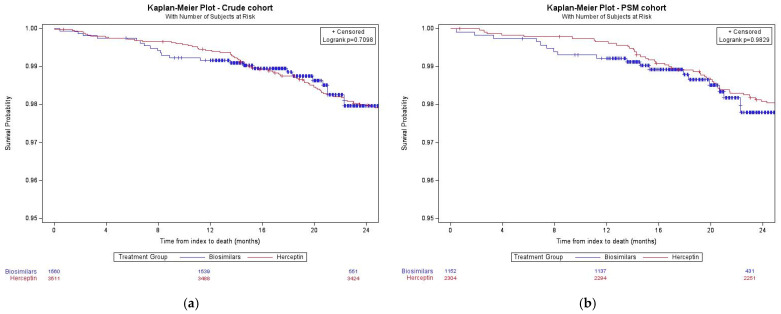
Survival curves stratified by treatment group: (**a**) is survival in the crude cohort and (**b**) is survival in the PSM cohort.

In the PSM cohort, no significant difference in OS (log-rank test *p* = 0.98) (Figure 3) or risk of mortality (HR: 1.29, 95% CI: 0.72–2.30, *p* = 0.39) was identified between treatment groups. The effectiveness results of the early treatment discontinuation analysis were consistent with those reported for the primary analysis (Figure S2).

## 4. Discussion

In this cohort study, we assessed the real-world safety and effectiveness of the implementation of neoadjuvant/adjuvant biosimilar trastuzumab compared to Herceptin among Ontario patients with HER2+ breast cancer. No differences in OS, ED visits leading to hospital admission, in-patient admission for CHF, or early treatment discontinuation were identified between treatment groups. We identified lower direct hospitalization among biosimilar trastuzumab compared to Herceptin patients. 

No difference in odds of in-patient hospital admission for CHF was identified between patients in the biosimilar trastuzumab compared to the Herceptin group. Clinical trials and a real-world study have similarly demonstrated no differences in cardiac safety, measured by left ventricular ejection fraction, between patients with HER2+ early-stage breast cancer receiving biosimilar trastuzumab compared to reference trastuzumab [11,12,15]. The results of our in-patient hospital admission for CHF analysis were consistent with those reported in the literature [11,12,15], though our findings may not be directly comparable, due to our use of healthcare service utilization data to measure cardiac safety.

The results of the early treatment discontinuation analysis suggest patients receiving biosimilar trastuzumab, compared to patients receiving Herceptin, did not experience increased toxicity events which would have required early stopping of trastuzumab treatment. These findings provide further support for similar real-world safety between biosimilar trastuzumab and Herceptin for neoadjuvant/adjuvant treatment of HER2+ breast cancer. 

The Kaplan–Meier curves overlapped, and no differences in the risk of mortality between treatment groups were identified. The findings of the effectiveness analysis are consistent with those reported in the literature comparing biosimilar trastuzumab and Herceptin [8,12,15]. We were unable to measure the median OS due to limited follow-up time.

A strength of this study is its large sample size. Additionally, we were able to include all Ontario patients receiving publicly funded biosimilar trastuzumab or Herceptin for neoadjuvant/adjuvant treatment of HER2+ breast cancer through the use of population-based real-world data. We were able to explore the comparability of biosimilar trastuzumab to Herceptin among patients with diverse demographic and clinical characteristics due to our use of real-world data, thereby increasing the generalizability of our study and study findings. The risk of confounding by indication was minimized due to the reason for patients receiving biosimilar trastuzumab or Herceptin being based on Ontario drug funding policies and not clinical factors affecting physician choices. 

This study is not without limitations. Despite the sample size, the expected good prognosis of patients with early-stage breast cancer together with the relatively short follow-up resulted in very low event rates (less than 5%) which severely limited the power of the study to detect clinically important differences. Therefore, the current survival findings are not yet conclusive, and further explorations should be conducted with more mature data. Specifically, future explorations with follow-up data of at least 5 years should be conducted to assess overall survival and relevant safety endpoints with more mature data. The findings from explorations with 5 years of follow-up data will be important to confirm the effectiveness and safety results reported in this study. Patients could not be randomized to receive biosimilar trastuzumab or Herceptin, limiting the comparability between the treatment groups. We attempted to mitigate issues of comparability between treatment groups by reducing the accrual window from 16 years to 5 years and applying PSM methods to balance baseline characteristics between treatment groups. Trends in cancer and non-cancer healthcare service utilization among patients may have been affected due to the timing of biosimilar trastuzumab being implemented a few months prior to the start of the COVID-19 pandemic. The influence of the COVID-19 pandemic on healthcare service utilization may have specifically affected patients starting biosimilar trastuzumab as their accrual period was during the COVID-19 pandemic while the historical comparator group had an accrual period prior to the COVID-19 pandemic. The reliability of ambulatory data, in particular, has been shown to be influenced by the COVID-19 pandemic [16,17]. This prevented explorations of ED visits not leading to hospitalization and the use of a validated algorithm for CHF which relies on ambulatory data in addition to in-patient hospital admission data. We were unable to control for the impact of the COVID-19 pandemic in our safety analyses due to data limitations. It is unlikely biosimilar trastuzumab leads to true reductions in toxicity events given the findings of the safety analyses between patients receiving biosimilar and reference trastuzumab reported elsewhere [7,8,9,10,11,12,15]. Regardless, observed trends in safety outcomes should be reviewed with caution. 

In this study, we demonstrated the real-world safety and effectiveness of biosimilar trastuzumab compared to Herceptin. Real-world studies provide opportunities to describe findings representative of real-world patients through their use of routinely collected administrative health data. A reliance on administrative data can limit the study cohorts, outcomes, and covariates included in real-world studies and how these parameters are defined. Still, real-world studies allow for comparisons of biosimilar and reference drugs among diverse patient groups. The evidence generated from real-world studies provides the potential to improve patient and clinician confidence in the implementation and use of biosimilar products.

## 5. Conclusions

Biosimilar trastuzumab demonstrated similar effectiveness and safety to Herceptin. A difference in the rate of ED visits leading to hospitalization was identified between treatment groups. Our findings may increase support for and confidence in the use of biosimilar trastuzumab among clinicians and patients. This study is additional evidence in support of the continued implementation of biosimilars to increase efficiencies and redirect funding resources to new, innovative technologies.

## Figures and Tables

**Table 1:** Baseline characteristics of crude and PSM cohorts.

		Crude Cohort			PSM Cohort		
		Herceptin	Biosimilar Trastuzumab	*p*-Value	Herceptin	Biosimilar Trastuzumab	Standardized Difference
		N = 3511	N = 1560		N = 2304	N = 1152	
Age at index, years	Mean ± SD	57.5 ± 12.3	57.9 ± 12.7	0.24	57.6 ± 11.9	57.2 ± 11.9	0.04
Sex, n (%)	Female	3495 (99.5)	1554 (99.6)	0.82	2304 (100.0)	1152 (100.0)	0
Rurality, n (%)	Rural	403 (11.5)	157 (10.1)	0.09	239 (10.4)	118 (10.2)	0
	Urban	3082 (87.8)	1384 (88.7)		2065 (89.6)	1034 (89.8)	
	Unknown	26 (0.7)	19 (1.2)		0 (0)	0 (0)	
Local Health Integration Networks, n (%)	Central	446 (12.7)	223 (14.5)	0.04	324 (14.1)	163 (14.2)	<0.01
	Central East	419 (11.9)	152 (9.7)		124 (5.4)	49 (4.3)	
	Central West	189 (5.4)	70 (4.5)		194 (8.4)	99 (8.6)	
	Champlain	380 (10.8)	147 (9.4)		69 (3.0)	31 (2.7)	
	Erie St. Clair	187 (5.3)	92 (5.9)		123 (5.3)	65 (5.6)	
	Hamilton Niagara Haldimand Brant	407 (11.6)	159 (10.2)		204 (8.9)	123 (10.7)	
	Mississauga Halton	267 (7.6)	121 (7.8)		249 (10.8)	131 (11.4)	
	North East	157 (4.5)	64 (4.1)		115 (5)	54 (4.7)	
	North Simcoe Muskoka	105 (3.0)	38 (2.4)		197 (8.6)	97 (8.4)	
	North West	44 (1.3)	21 (1.4)		286 (12.4)	128 (11.1)	
	South East	137 (3.9)	68 (4.4)		85 (3.7)	56 (4.3)	
	South West	307 (8.7)	169 (10.8)		195 (8.5)	95 (8.3)	
	Toronto Central	271 (7.7)	131 (8.4)		31 (1.4)	12 (1.0)	
	Waterloo Wellington	169 (4.8)	86 (5.5)		108 (4.7)	49 (4.3)	
Days from Dx date to index	Mean ± SD	116.7 ± 44.8	101.0 ± 44.7	<0.01	108.7 ± 38.3	104.8 ± 39.0	0.08
Ontario Marginalization Index score quintiles, n (%)	1 (lowest	827 (23.6)	399 (25.6)	0.14	557 (21.2)	290 (25.1)	0
	2	423 (12.1)	211 (13.5)		302 (13.1)	151 (13.1)	
	3	774 (22.1)	328 (21.0)		533 (23.1)	250 (21.7)	
	4	630 (17.9)	281 (18.0)		393 (17.1)	221 (19.2)	
	5 (highest)	808 (23.0)	316 (20.3)		519 (22.5)	240 (20.8)	
	Missing	49 (1.4)	25 (1.6)		0 (0)	0 (0)	
Stage, n (%)	I	1397 (39.8)	794 (50.9)	<0.01	1144 (49.7)	572 (49.7)	0
	II	1491 (42.5)	558 (35.8)		858 (37.2)	429 (37.2)	
	III	623 (17.7)	208 (13.3)		302 (13.1)	151 (13.1)	
Estrogen–progesterone receptor status, n (%)	Positive	2453 (69.9)	945 (60.6)	<0.01	1538 (66.7)	769 (66.7)	0
	Both negative/ indeterminate	1058 (30.1)	615 (39.4)		766 (33.3)	383 (33.3)	
Charlson Comorbidity Index score, n (%)	No hospitalization	NR *	NR *	0.08	0 (0)	0 (0)	<0.01
	0	3034 (86.4)	1375 (88.1)		2031 (88.2)	1025 (88.9)	
	1	374 (10.7)	155 (9.9)		225 (9.8)	110 (9.6)	
	2+	103 (2.9)	30 (1.9)		48 (2.1)	17 (1.5)	
History of prior breast cancer, n (%)	Yes	161 (4.6)	76 (4.9)	0.05	92 (4.0)	51 (4.4)	0.02
History of prior non-breast cancer, n (%)	Yes	304 (8.7)	147 (9.4)	0.03	210 (9.1)	102 (8.9)	<0.01
CHF within 3 years prior to index date, n (%)	Yes	30 (0.9)	17 (1.1)	0.09	16 (0.7)	8 (0.7)	0
Receipt of trastuzumab with respect to breast resection date, n (%)	Neoadjuvant	1152 (32.8)	751 (48.1)	<0.01	857 (37.2)	487 (42.3)	0
	Adjuvant	2359 (67.2)	809 (51.9)		1447 (62.8)	665 (57.7)	
Receipt of systemic chemotherapy with respect to breast resection date, n (%)	Neoadjuvant	1183 (33.7)	769 (49.3)	<0.01	865 (37.5)	496 (43.1)	0
	Adjuvant	2328 (66.3)	791 (50.7)		1439 (62.5)	656 (56.9)	
History of RT to breast tissue for prior breast cancer, n (%)	Yes	87 (2.5)	52 (3.3)	0.02	53 (2.3)	34 (2.9)	0.04
RT to breast tissue between index and 1 year after breast cancer Dx, n (%)	Yes	1876 (53.4)	787 (50.5)	<0.01	1249 (54.2)	622 (54.0)	<0.01
Systemic chemotherapy regimen, n (%)	Anthracycline-based only	62 (1.8)	8 (0.5)	<0.01	8 (0.4)	NR *	0
	Anthracycline taxane	2198 (62.6)	867 (55.6)		1502 (65.2)	755 (65.6)	
	Platinum taxane	295 (8.4)	235 (15.1)		186 (8.1)	93 (8.1)	
	1st-generation CMF	14 (0.4)	NR *		0 (0)	0 (0)	
	2nd-generation taxane	371 (10.6)	125 (8.0)		182 (7.9)	91 (7.9)	
	APT	571 (16.3)	325 (20.8)		426 (18.5)	213 (18.5)	

APT, paclitaxel and trastuzumab; CHF, congestive heart failure; CMF, cyclophosphamide, methotrexate, fluorouracil; Dx, diagnosis; RT, radiation; SD, standardized deviation. * Cells with <6 were not reported (NR). To prevent back-calculation, numbers were combined with Score 1 for baseline characteristic Charlson Comorbidity Index score and anthracycline taxane for baseline characteristic systemic chemotherapy regimen.

**Table 2:** Frequencies, odds ratios, and rate ratios of safety events for crude and PSM cohorts.

		Crude Cohort			PSM-Adjusted Cohort		
		Herceptin	Biosimilar Trastuzumab	*p*-Value	Herceptin	Biosimilar Trastuzumab	*p*-Value
		N = 3511	N = 1560		N = 2304	N = 1152	
Treatment-related death	n (%)	NR *	NR *	0.43	0 (0)	NR *	0.33
	OR (95% CI)	0.44 (0.03–7.11)		0.57	-		-
Direct hospital admission	n (%)	965 (27.5)	360 (23.0)	<0.01	647 (28.1)	263 (22.8)	<0.01
	RR (95% CI)	0.92 (0.82–1.04)		0.21	0.85 (0.74–0.98)		0.03
ED visit leading to hospitalization	n (%)	428 (12.2)	168 (10.8)	0.15	266 (11.6)	121 (10.5)	0.36
	RR (95% CI)	0.99 (0.82–1.21)		0.98	0.96 (0.77–1.20)		0.72

CI, confidence interval; ED, emergency department; OR, odds ratio; RR, rate ratio; * Cells with <6 were not reported (NR).

**Table 3:** Frequency and odds ratio of in-patient hospital admission for CHF for crude and adjusted cohorts among patients with minimum 1 year of follow-up time.

		Crude Cohort			PSM-Adjusted Cohort		
		Herceptin	Biosimilar Trastuzumab	*p*-Value	Herceptin	Biosimilar Trastuzumab	*p*-Value
		N = 3488	N = 1539		N = 2294	N = 1137	
In-patient hospital admission for CHF	n (%)	27 (0.77)	15 (0.97)	0.50	18 (0.78)	10 (0.88)	0.77
	OR (95% CI)	1.26 (0.67–2.38)		0.47	1.12 (0.52–2.44)		0.77

CHF, congestive heart failure; CI, confidence interval; OR, odds ratio.

**Table 4:** Frequency and odds ratio of early treatment discontinuation for crude and adjusted cohorts among patients receiving a treatment regimen of one dose every 3-week treatment cycle.

		Crude Cohort			PSM-Adjusted Cohort		
		Herceptin	Biosimilar Trastuzumab	*p*-Value	Herceptin	Biosimilar Trastuzumab	*p*-Value
		N = 2792	N = 1013		N = 1588	N = 794	
Early treatment discontinuation	n (%)	694 (24.9)	252 (24.9)	0.99	396 (24.9)	185 (23.3)	0.38
	OR (95% CI)	0.99 (0.85–1.18)		0.99	0.91 (0.75–1.12)		0.38

CI, confidence interval; OR, odds ratio.

## Data Availability

Ontario Health is prohibited from making the data used in this research publicly accessible if it includes potentially identifiable personal health information and/or personal information as defined in Ontario law, specifically the Personal Health Information Protection Act (PHIPA) and the Freedom of Information and Protection of Privacy Act (FIPPA). Upon request, data de-identified to a level suitable for public release may be provided. Parts of this material are based on data and information provided by Ontario Health (Cancer Care Ontario) [and include data received by Ontario Health (Cancer Care Ontario) from the Canadian Institute for Health Information (CIHI) and ICES]. The opinions, reviews, views, and conclusions reported in this publication are those of the authors and do not necessarily reflect those of Ontario Health (Cancer Care Ontario) [CIHI and/or ICES]. No endorsement by Ontario Health (Cancer Care Ontario) [CIHI and/or ICES] is intended or should be inferred.

## Supplementary Materials

The following supporting information can be downloaded at: https://www.mdpi.com/article/10.3390/curroncol31030124/s1, Figure S1. Kaplan-Meier survival curves by treatment group in the crude cohorts for accrual windows from 2005–2021 (Box A), 2011–2021 (Box B), and 2016–2021 (Box C). Figure S2. Kaplan-Meier survival curves of the early treatment discontinuation analysis which excluded patients with a record of switching to TDM-1 for the crude cohort (Box A) and PSM cohort (Box B).
