# Supplementary material for: Comparative Effectiveness and Safety of Trastuzumab Biosimilars to Herceptin for Adjuvant Treatment of HER2+ Breast Cancer
Source: Curr Oncol. 2024 Mar 21;31(3):1633–44. doi: 10.3390/curroncol31030124 (PMC10969053; doi:10.3390/curroncol31030124)
Supplement: Supplementary file 1 [file curroncol-31-00124-s001.zip › curroncol-2890407-supplementary.pdf]

Supplemental materials

Figure S1. Kaplan-Meier survival curves by treatment group in the crude cohorts for accrual windows from 2005-2021 (Box A), 2011-2021 (Box B), and 2016-2021 (Box C).

Figure S2. Kaplan-Meier survival curves of the early treatment discontinuation analysis which excluded patients with a record of switching to TDM-1 for the crude cohort (Box A) and PSM cohort (Box B).

The original design of this study was to include all patients with a record of Herceptin from the start of funding for adjuvant treatment of patients with HER2+ breast cancer in 2005. The initial cohort as shown in Figure 1 captured patients from the OCR and NDFP from 2005 to 2021. The Kaplan Meier survival curves for accrual periods from 2005 to 2021, 2011-2021, and 2016-2021 were reviewed to aid in our decision to shorten the accrual period to support comparability of the treatment groups.

Box A

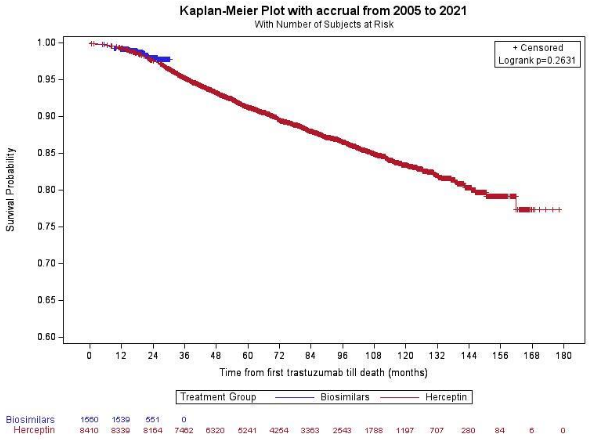

Box B

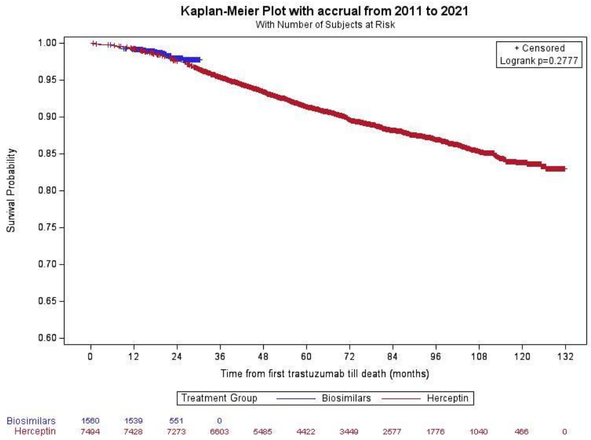

Box C

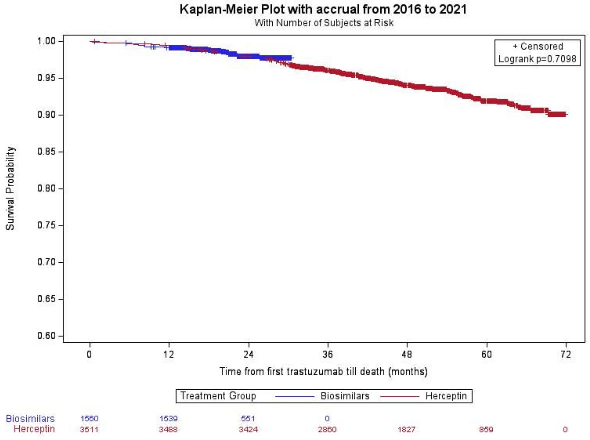

Figure S1. Kaplan-Meier survival curves by treatment group in the crude cohorts for accrual windows from 2005-2021 (Box A), 2011-2021 (Box B), and 2016-2021 (Box C).

Box A Box B

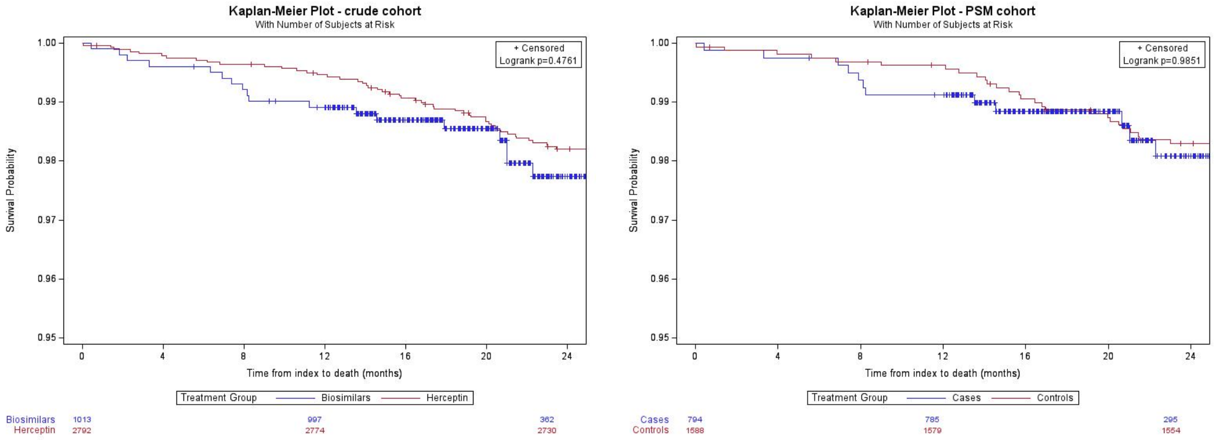

Figure S2. Kaplan-Meier survival curves of the early treatment discontinuation analysis which excluded patients with a record of switching to TDM-1 for the crude cohort (Box A) and PSM cohort (Box B).
